# Editorial: The COVID-19 pandemic and social cohesion across the globe

**DOI:** 10.3389/fsoc.2023.1182452

**Published:** 2023-05-10

**Authors:** Mandi M. Larsen, Georgi Dragolov, Jan Delhey

**Affiliations:** ^1^Bremen International Graduate School of Social Sciences (BIGSSS), Constructor University, Bremen, Germany; ^2^School of Business, Social and Decision Sciences, Constructor University, Bremen, Germany; ^3^Department of Sociology, Institute for Social Sciences, Otto-von-Guericke University Magdeburg, Magdeburg, Germany

**Keywords:** coronavirus pandemic, COVID-19, social cohesion, existential insecurity, social relations, social trust, institutional trust, solidarity

## Introduction

The COVID-19 pandemic has brought about a constellation of health, social, economic, and political crises, drastically affecting the lives of people across the globe. Governments in many countries implemented dramatic public health measures in order to prevent the spread of the virus (Fong et al., 2020). Unprecedented restrictions were imposed on individual mobility which brought public life to a standstill in many places, with constraints placed on businesses, places of education, transportation, as well as on leaving one's own home. These social distancing mandates imposed by governments required the collective action of individuals to mitigate the spread of the highly infectious virus, especially prior to the availability of vaccines. “More than ever we depend[ed] on fellow citizens to behave responsibly, and on institutional actors to make the right decisions” (Delhey et al., 2021, p. 3).

Moreover, social inequalities—particularly along income, race, ethnicity, and gender lines—influenced which groups were most affected by the pandemic with regards to infection as well as the pandemic's social and economic consequences. This dramatic societal disruption resulted in initial workplace shifts and job loss, temporary disruption in financial assistance provided by social welfare institutions, and overall deterioration in wellbeing (Brodeur et al., 2021). Whoever belonged to a vulnerable group before the pandemic (e.g., the poor, the unemployed, ethnic or racial minorities), likely has fewer resources to cope with these continuing challenges, so that inequalities might even widen (Jewett et al., 2021).

For these reasons, the pandemic and its socioeconomic repercussions highlight the vital importance of social cohesion, as always in times of deep crises or great catastrophes (Townshend et al., 2015). Social cohesion is often described as the glue that holds society together, as an “attribute of a collective, indicating the quality of collective togetherness” (Schiefer and van der Noll, 2017, p. 592). Whether societies will be living with its consequences for the longer term or will soon be able to overcome them, the COVID-19 pandemic offers a unique opportunity to examine from a sociological perspective how a sudden and profound threat to existential security impacts social cohesion. Have societies “come together” to withstand the shared threat as posited, for example, by the “rally-round-the-flag” thesis (Bol et al., 2021; Kritzinger et al., 2021)? Or are they “coming apart” (Borkowska and Laurence, 2021), as the question of how to respond to the crisis has become increasingly divisive?

In order to design long-term strategies for dealing with the social consequences of the pandemic, a strong foundation of innovative scientific knowledge covering a broad spectrum of societies and perspectives over an extended period of time is necessary. This has been the aim of the present Research Topic of *Frontiers in Sociology*. It called specifically for contributions on how the pandemic has affected various aspects of social cohesion, such as “resilient social relations, positive emotional connectedness between its members and the community, and a pronounced focus on the common good” (Dragolov et al., 2016, p. 6). Taken together, the quantitative empirical papers published here (see Table 1) contribute to the understanding of social relations, attitudes toward migration, interpersonal trust, ideological polarization, a shared understanding of reality, provision of instrumental help, compliance with containment measures, and poverty during the pandemic. An additional theoretical contribution by Posocco and Watson argues for the necessity of reimagining “a new world order based on cooperation, coordination, and solidarity between nation-states” (p. 1) in times of crisis like the pandemic. Collectively, the evidence presented in this Research Topic lays significant groundwork for a more contextualized understanding of the social impact of the pandemic across the globe.

**Table 1:** Empirical global perspectives on the COVID-19 pandemic and social cohesion.

**Authors**	**Title**	**Country**	**Population**	**Research design**	**Topic of research**
Bergmann et al.	The impact of the COVID-19 pandemic on the provision of instrumental help by older people across Europe	27 European countries and Israel	Adults, aged 50+ years	Panel survey (2020–2021); 2 waves; *N* = 45,000+	Provision of instrumental help
Castillo et al.	Social cohesion and attitudinal changes toward migration: A longitudinal perspective amid the COVID-19 pandemic	Chile	Adults, aged 18+ years	Panel survey (2016–2021); 5 waves; *N* = 1,611	Attitudes toward migration
Dochow-Sondershaus	Ideological polarization during a pandemic: Tracking the alignment of attitudes toward COVID containment policies and left-right self-identification	Austria	Adults and adolescents, aged 14+ years	Panel survey (2020–2021); 24 waves; *N* = 1,500	Ideological polarization
Eichhorn et al.	Reality bites: An analysis of Corona deniers in Germany over time	Germany	Adults and adolescents, aged 16+ years	Panel survey (2020–2021); 2 waves; *N* = 1,280	Shared understanding of reality
Nahkur and Kutsar	The change in children's subjective relational social cohesion with family and friends during the COVID-19 pandemic: A multinational analysis	Albania, Algeria, Bangladesh, Belgium, Chile, Estonia, Finland, Germany, Indonesia, Israel, Italy, Romania, Russia, South Korea, Spain, Taiwan, Turkey, and Wales	Children, primarily aged 9–13 years	Cross-sectional survey (2021); *N* = 20,000+	Social relations
Petersen et al.	The burdens of poverty during the COVID-19 pandemic	Germany	Adults, aged 25+ years	Prospective cohort survey (2020–2021); 2 waves; *N* = 8,100	Poverty
Schröder et al.	Trust and compliance: Milieu-specific differences in social cohesion during the COVID-19 pandemic in Germany	Germany	Adults and adolescents, aged 15+ years	Cross-sectional survey (2020); *N* = 589	Trust; Compliance
Tatarko et al.	Social capital and the COVID-19 pandemic threat: The Russian experience	Russia	Adults, aged 18+ years	Cross-sectional survey (2020); *N* = 500	Social relations; Institutional trust

## Longitudinal and cross-sectional research

With the help of longitudinal data, many of the studies included in this Research Topic were able to illustrate how the pandemic has shifted over time since its initial waves. Particularly impressive in this regard is the Austrian Corona Panel Project (ACPP; Kittel et al., 2020), which Dochow-Sondershaus used to track attitude shifts related to COVID-19 containment measures over the course of more than a year according to individual ideological self-identification. About 1,500 respondents were surveyed a total of 24 times between March 2020 and July 2021, often on a weekly basis. This allowed Dochow-Sondershaus to place the trajectories of ideological groups in the context of key time points of the pandemic in Austria (e.g., the first lockdown, introduction of mask mandates, and so on), illustrating the dynamics of ideological divergence and convergence of attitudes regarding pandemic containment measures.

The Values in Crisis (VIC) panel survey project was fielded in Germany and the United Kingdom in order to study how citizens' moral value orientations react to the social disruption caused by the pandemic. By analyzing VIC data from nearly 1,300 respondents in Germany in the first months of the pandemic (April-May 2020) and then again in the early months of the following year (February–March 2021), Eichhorn et al. drew conclusions about whether those who supported pandemic-related conspiracy beliefs at the beginning of the pandemic were the same as those who held these beliefs later on. This enabled the authors to identify socio-demographic and attitudinal profiles where pandemic conspiracy beliefs became ingrained over time.

Three studies in this Research Topic made use of well-established longitudinal survey projects which were initiated well before Corona. Bergmann et al. analyzed two waves of the Survey of Health, Aging and Retirement in Europe (SHARE) Corona Survey (Börsch-Supan, 2022a,b) involving 45,000+ older adults. In doing so, the authors examined individual changes in providing and receiving instrumental help between the first summer of the pandemic and about 1 year later. Similarly, Petersen et al. used two waves of the Gutenberg COVID-19 Study, a population-representative, prospective cohort study, which built on the original Gutenberg Health Study in the Mainz and Mainz-Bingen areas of Germany (Wild et al., 2012). In doing so, the authors identified respondents at-risk of living in poverty and compared their outcomes at the second time point with regards to economic impacts and psychosocial stressors of the pandemic. Finally, instead of adding on pandemic-dedicated waves as the previous two studies did, Castillo et al. tracked changes in attitudes toward migrants over four waves of data collection prior to the pandemic (2016–2019) and one wave in the midst of it (2021) from the Chilean Longitudinal Social Survey (ELSOC; Reproducible Research Centre for Social, Conflict and Cohesion Studies, COES, 2022) to assess the impact of the pandemic on these attitudes.

Likewise, the cross-sectional studies featured make their own valuable contributions to the literature, such as developing an empirical typology of social milieus (Schröder et al.), being one of the first studies to examine children's relational social cohesion with large scale, multinational quantitative research (Nahkur and Kutsar), and offering insights into social relations in Russia (Tatarko et al.).

## Research across the globe

One of the primary aims of the Research Topic was to highlight research from a wide range of countries, regions, and cultures in order to broaden our understanding of the effects of this truly global pandemic on social cohesion. The papers in this Research Topic contribute to this aim in a variety of manners. A number of country-specific studies offer unique national perspectives on Austria (Dochow-Sondershaus), Chile (Castillo et al.), Germany (Eichhorn et al.; Petersen et al.; Schröder et al.), and Russia (Tatarko et al.). Particularly when combined with a longitudinal study design (Castillo et al.; Dochow-Sondershaus; Eichhorn et al.; Petersen et al.), these studies offer intensive examinations of the respective country.

These national case studies are complemented by two multinational studies that add important comparative insights. Bergmann et al.'s analysis of the SHARE Corona Survey used full probability samples from 27 European countries and Israel, offering internationally comparable representative data for populations aged 50 and above; these data allowed them to take into consideration the different national contexts with regards to the varying policy responses to the pandemic, as well as levels of severity at various time points. Similarly, as part of the International Children's Worlds COVID-19 Supplement Survey, Nahkur and Kutsar analyzed cross-sectional data of 20,000+ children (primarily 9–13 years of age) collected in 2021 from 18 countries across Europe, Asia, and North Africa in their investigation of the impact of the pandemic on children's relational social cohesion with family and friends.

## Populations and sub-populations

The majority of empirical studies included in this Research Topic target the “typical” adult population (see Table 1). Two of the papers, however, present unique generational perspectives. At the beginning of the pandemic in particular, the elderly were perceived as being in need of protection and provision of instrumental support, but Bergmann et al. take a closer look at the changing patterns of how individuals aged 50+ in Europe have provided help to others during the pandemic. Nahkur and Kutsar offer another point of view, arguing that children are both embedded in the social networks of their families and creating their own networks. Thus, given the widespread school closures and other lockdown measures across the globe, their relational patterns with friends and family were altered, with potential impact on their social development and mental health.

## Social cohesion in the pandemic: substantial insights

In the remainder of this editorial, we discuss research insights along the three main components of the Bertelsmann Social Cohesion Radar (Dragolov et al., 2016) mentioned in the introduction. Several papers speak to the first component, resilient social relations, which involves the horizontal relationships of individuals, and comprises intact social networks, trust in others, and acceptance of diversity. Contributions to this Research Topic share clear indications of weakened social relations, though the picture is more complex than previously thought. Nahkur and Kutsar demonstrate that social distancing measures during the pandemic affected children differently depending on the severity of measures experienced. Across all 18 countries studied, about one in 10 reported feeling as if their social relationships had considerably decreased (and about one in four reported this in Germany, Turkey, and Bangladesh). In Russia, Tatarko et al. find evidence of weakened social ties with neighbors and fellow citizens, but unchanged or intensified ties with family, colleagues, and friends. The authors speculate that these associations are a reaction to threat and isolation, with people worrying about their next of kin and contacting them more often in isolation, while contacting weaker ties even less than before.

In order to gain a more nuanced understanding of interpersonal trust during the pandemic in Germany, Schröder et al. propose a new model of social milieus which combine socioeconomic status and basic human values of social groups. Their results from the first wave of COVID-19 indicate greater heterogeneity than would be expected based on the “rally-round-the-flag” thesis. The authors find the highest levels of trust in a milieu in the lower socioeconomic class with socially focused values, and the lowest trust in the upper-middle class milieu with personally focused values.

With regards to acceptance of diversity, Castillo et al. argue that in the past, migrants have been seen as potential carriers of disease and potential threats (Kraut, 2010), even when evidence indicated otherwise. Castillo et al. indeed demonstrate that Chileans perceive migrants more negatively after the pandemic, especially lower-educated Chileans and those who live in neighborhoods with an increasing number of migrants.

This Research Topic also aimed to highlight pandemic-induced shifts in feelings of connectedness, the second main component of social cohesion. This component taps the emotional and attitudinal attachment of citizens toward the wider institutional framework. Since government pandemic containment strategies had not previously been strongly associated with an ideological or partisan identity, Dochow-Sondershaus took advantage of the unique opportunity offered by the COVID-19 pandemic to examine polarizing trends over time in Austria according to ideological self-identity. While all of the various ideological groups generally perceived the government's policies for containing COVID-19 as appropriate at first, this shifted over time. Eventually, the positions of right-wing and left-wing identifiers solidified, with the former finding the policies “too extreme.” However, toward the end of the study period (December 2020-February 2021), Dochow-Sondershaus does note a certain degree of convergence toward views of containment policies being a bit “too extreme” among all groups. During this time period, no lockdowns were taking place in Austria, and there were some signs of normalization (e.g., widely available self-tests and rising vaccination rates) that left the impression that the pandemic had become politically manageable. The study by Eichhorn et al. on Corona deniers in Germany provides evidence that considering Corona a hoax is deeply intertwined with low political trust and low trust in “mainstream” media. The authors attest to an extreme attitude profile especially to the—fortunately, not very large in Germany—camp of “consistent deniers” who held this opinion in 2020 and 2021.

Several contributions to this Research Topic also dealt with the third building block of social cohesion—the focus on the common good. This cohesion component highlights, in particular, the importance of context for respect for social rules, as well as for solidarity and helpfulness. In their examination of concerned compliance with governmental pandemic measures based on social milieus in Germany, Schröder et al. find that milieus with socially focused values demonstrate high concerned compliance, whereas those that held self-enhanced and personally focused values demonstrate low concerned compliance. In a similar vein, Eichhorn et al. provide evidence that people who endorse conformity more strongly are significantly less likely to consider the pandemic a hoax. In that sense, being “other-oriented” in a positive way contributes to societal cohesion, also under the pandemic condition. From the angle of intergenerational functional solidarity, Bergmann et al. discovered that help from adult children (aged 50+) to elderly parents strongly increased in the first phase of the pandemic, while support from elderly parents to their adult children decreased during this phase, especially in countries that faced the largest challenges in 2020 due to the pandemic. Moreover, provision of instrumental help by older adults to people outside of the family was common at the start of the pandemic, but strongly decreased by 2021. The contribution by Petersen et al. reminds us that the pandemic led to increased risks of poverty and psychological stress, despite considerable solidarity among people. This is an example of the limits of what cohesion can achieve in times of a deep crisis.

## Conclusions

The authors who contributed to this Research Topic have cumulatively begun building a foundation of innovative scientific knowledge on social cohesion in the COVID-19 pandemic. With their investigations across time and space, the contributions add a great degree of context to the current research by illustrating the ever-changing landscape of the pandemic and its impact. In short, they provide no straightforward answer to the question of whether societies are “coming together” or “coming apart.” Instead, they offer a body of evidence demonstrating the necessity of considering intergenerational relationships, societal differences, and relevant phases of the pandemic and their related containment measures. Understanding this complexity appears to be the key to developing long-term strategies for dealing with the social consequences of this and future pandemics.

## Author contributions

All authors listed have made a substantial, direct, and intellectual contribution to the work and approved it for publication.
